# Patients’ Use of Social Media for Diabetes Self-Care: Systematic Review

**DOI:** 10.2196/14209

**Published:** 2020-04-24

**Authors:** Abdelaziz Elnaggar, Van Ta Park, Sei J Lee, Melinda Bender, Lee Anne Siegmund, Linda G Park

**Affiliations:** 1 Department of Community Health Systems School of Nursing University of California, San Francisco San Francisco, CA United States; 2 Division of Geriatrics School of Medicine University of California, San Francisco San Francisco, CA United States; 3 Department of Family Health Care Nursing School of Nursing University of California, San Francisco San Francisco, CA United States; 4 Office of Nursing Research and Innovation Cleveland Clinic Cleveland, OH United States

**Keywords:** social media, diabetes mellitus, peer group, self-care, systematic review

## Abstract

**Background:**

Patient engagement with diabetes self-care is critical to reducing morbidity and mortality. Social media is one form of digital health that is available for diabetes self-care, although its use for peer-to-peer communication has not been systematically described, and its potential to support patient self-care is unclear.

**Objective:**

The primary aim of this systematic review was to describe the use of social media among patients (peer-to-peer) to manage diabetes and cardiovascular disease (CVD). The secondary aim was to assess patients’ clinical outcomes, behavioral outcomes, quality of life, and self-efficacy resulting from peer-to-peer social media use.

**Methods:**

We conducted a literature search in the following databases: PubMed, EMBASE, Web of Science, CINAHL, and PsycINFO (January 2008 through April 2019). The inclusion criteria were quantitative studies that included peer-to-peer use of social media for self-care of diabetes mellitus (with all subtypes) and CVD, including stroke.

**Results:**

After an initial yield of 3066 citations, we selected 91 articles for a full-text review and identified 7 papers that met our inclusion criteria. Of these, 4 studies focused on type 1 diabetes, 1 study included both type 1 and 2 diabetes, and 2 studies included multiple chronic conditions (eg, CVD, diabetes, depression, etc). Our search did not yield any individual studies on CVD alone. Among the selected papers, 2 studies used commercial platforms (Facebook and I Seek You), 3 studies used discussion forums developed specifically for each study, and 2 surveyed patients through different platforms or blogs. There was significant heterogeneity in the study designs, methodologies, and outcomes applied, but all studies showed favorable results on either primary or secondary outcomes. The quality of studies was highly variable.

**Conclusions:**

The future landscape of social media use for patient self-care is promising. However, current use is nascent. Our extensive search yielded only 7 studies, all of which included diabetes, indicating the most interest and demand for peer-to-peer interaction on diabetes self-care. Future research is needed to establish efficacy and safety in recommending social media use among peers for diabetes self-care and other conditions.

## Introduction

### Background

Diabetes is one of the most prevalent chronic conditions in the United States and worldwide [1-3], associated with high morbidity and mortality, mainly as a result of complications from cardiovascular disease (CVD) [4-8]. In 2016, the World Health Organization estimated that diabetes was the seventh leading cause of death [9]. Evidence indicates that managing blood glucose and diabetes risk factors (including CVD) can prevent or delay mortality because of CVD by 33% [10-13]. Patient engagement is critical to successfully managing diabetes and thereby reducing morbidity and mortality [14,15].

Self-care has been described as a vital component in diabetes prevention and management in addition to other chronic conditions such as CVD [16-19]. Defined as a “naturalistic decision‐making process addressing both the prevention and management of chronic illness” [16], self-care for chronic disease is a complex, multi-factorial endeavor with few effective intervention strategies to help patients manage their conditions [20]. Patients spend very little time each year with their providers and therefore need to independently build skills, knowledge, and motivation to improve individual outcomes. Several meta-analyses and reviews of multiple self-care intervention trials found lifestyle modification programs were more effective than usual care in improving clinical outcomes for diabetes and CVD [21-23].

Despite the known benefits, patients face many barriers in meeting the necessary lifestyle changes involved in self-care, including depression, poor self-efficacy, and cognitive decline [16]. Given the exponential rise in digital technology use among all age groups in the United States [24], mobile technologies are now frequently employed with lifestyle interventions to promote prevention, management, and self-care of chronic diseases [25-27]. Other technology-based programs such as telehealth and home-based rehabilitation have been successful for older patients and reflect their ability to adapt to the use of technology to support their health [28-30].

Peer-to-peer engagement [31], which is communicating with other people experiencing the same chronic condition to learn more about controlling and managing their condition, was found helpful to overcome some of these barriers [16] and has been shown to facilitate self-care, resulting in improved health behaviors [32]. Peer-to-peer communication through engagement on social media offers a convenient venue that is easily accessible for addressing patients’ educational needs and providing real-time interaction with others who share many of the challenges in disease management [33]. In a scoping review of social media use between patients and caregivers, researchers found that social media was used to facilitate self-care in 77.1% (219/284) studies identified. Among these studies, the majority of conclusions were positive about social media use [34]. Although younger age and ease with technology use have been shown to affect the likelihood of using social media for disease-related support [35], the number of older adults who engage in social media has continued to climb and offers significant potential to affect self-care [24]. In addition, more capable social media users have recognized the potential for providing support to others who are managing chronic conditions [35].

Innovative strategies and effective interventions are required to improve self-care and health outcomes for patients with diabetes and CVD. A recent systematic review found supplementing usual health care services using social media platforms can satisfy patients’ social support needs with managing their CVD, which health providers cannot easily accommodate [36]. Therefore, leveraging social media may be a viable strategy to help improve self-care for diabetes. Understanding how patients use social media to manage their chronic disease is a first step in validating social media platforms as a potentially effective intervention strategy to provide peer-to-peer support and improve diabetes self-care.

### Study Aims

The primary aim of this systematic review was to summarize the available evidence on the peer-to-peer use of social media for managing diabetes. A secondary aim was to assess patients’ clinical outcomes, behavioral outcomes (ie, self-care and patient activation), quality of life, and self-efficacy resulting from patients’ social media use.

## Methods

### Overview

In this systematic review, we conducted a comprehensive search to capture all of the relevant quantitative studies that were published on the use of a social media platform as a communication tool between patients (peer-to-peer) on health-related topics pertaining to diabetes and CVD self-care. The outcome of interest included any change in clinical outcomes, behavioral outcomes, quality of life, and self-efficacy in participating individuals who used social media for peer-to-peer communication. This systematic review was conducted using the Preferred Reporting Items for Systematic Reviews and Meta-Analysis guidelines [37]. The protocol of this review was registered on the International Prospective Register of Systematic Reviews on November 13, 2018, using the same name as this study’s review title.

The inclusion criteria included quantitative studies that addressed the use of social media as a communication tool between patients (ie, not between patients and providers). An 11-year interval (January 2008 to April 2019) was used to search for eligible studies as most studies with social media began in the late 2000s [38]. All US and international studies were included if they were available in the English language. We included studies that provided blogs, chats, and discussion forums from their Web-based platforms, but we excluded studies that were solely Web-based interventions (eg, education-based without interactions between participants). We limited our paper to describe the peer-to-peer use of social media and did not include studies describing the effect of health care provider-to-patient interactions on social media. We also excluded articles that did not mention which disease was studied. We excluded studies that were duplicates, book chapters, systematic or meta-analysis reviews, qualitative studies, editorials, and meeting abstracts. No studies were excluded on the basis of quality.

A systematic methodology was developed to capture all the relevant data from the selected articles. We ensured our included studies had a clear research question on the basis of population, intervention, comparator, outcomes, and study design criteria (Textbox 1) [39]. This paper presents a narrative synthesis as it was not possible to pool results for a meta-analysis.

**Outline of research questions on the basis of the population, intervention, comparator, outcomes, and study design criteria (PICOS framework).**

Population:

- Patients with diabetes

Intervention:

- Use of all social media platforms (eg, discussion forum, blogs, microblogs, and group chatting) for peer-to-peer communication for health-related reasons including support, advice, and education

Comparator:

- Patients receiving the same sort of treatment without social media exposure
- No comparator

Outcome:

- Clinical outcomes (eg, biological measures)
- Behavioral outcomes (eg, self-care and patient activation)
- Quality of life and self-efficacy

Study design:

- Randomized controlled trials
- Cohort
- Cross-sectional

### Search Strategy

The search terms were developed on the basis of our research question with the assistance of a health sciences librarian. The selected terms were intended to capture studies that used the most popular social media platforms in all major languages. These terms were adjusted to fit each database to avoid missing any articles (Multimedia Appendix 1). The literature search was conducted in PubMed, EMBASE, Web of Science (including all the databases included in it), CINAHL, and PsycINFO to identify potential articles. We then conducted a manual review of published articles and their bibliographies to assess eligibility for inclusion. In addition, we conducted a hand search of possible relevant articles in the *Journal of Medical Internet Research* and *JMIR Diabetes*.

### Study Selection

Initial screening of the studies was done by 2 independent reviewers (AE and MB). Primary screening and data extraction were done using the Cochrane Covidence primary screening and data extraction tool to import all the search results from databases followed by preliminary screening, which included titles and abstracts. If the preliminary screening of the abstract was not conclusive, the full text was screened (AE, MB, and VP). On the basis of the abovementioned criteria, studies were selected for a full-text review, with disagreements resolved by 2 other reviewers (LP and VP) who assessed the eligibility of the studies and approved the final selection of all included studies.

### Data Extraction and Analysis

We developed data extraction guidelines. One reviewer (AE) performed data extraction for each eligible article, which was subsequently verified by a second reviewer (MB). The following variables were extracted from the selected studies: name of the first author, year of publication, country, target condition and age of participants, study design and sample size, exposure or intervention, form of social media and purpose, outcome measures, and results. We conducted a descriptive analysis with a summary of the studies.

## Results

### Study Characteristics

**Figure 1:**
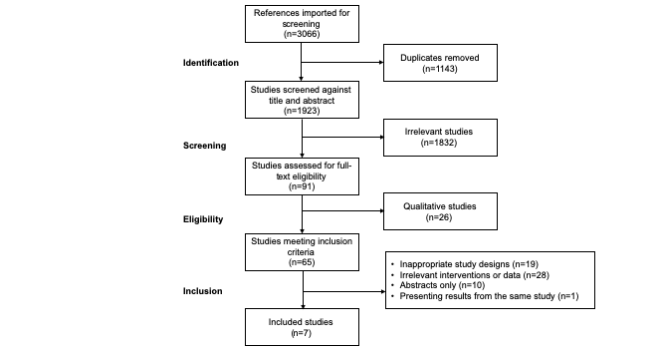
Flow chart of study selection process.

The initial database search applying our terms yielded 3066 citations. After removing duplicates, the remaining 1923 titles and abstracts were screened. On the basis of the inclusion and exclusion criteria, 91 articles were identified as eligible for a full-text review. Of these 91 articles, 84 did not meet the criteria and were eliminated as displayed in Figure 1, leaving 7 studies for inclusion that were related to diabetes and multiple chronic conditions, including CVD. We did not identify any studies focused on CVD alone.

The 7 selected studies for this review included 1 pilot randomized controlled trial (RCT) [40], 1 prospective cohort study [41], 3 cross-sectional studies [42-44], and 2 hybrid cross-sectional/cohort studies [45,46]. Of these, 1 study used Facebook [46], 1 study used a chat line platform [41], 3 studies used discussion forums that were developed specifically for each study [40,43,45], and 2 studies used surveys to assess the use of social networking sites/blogs [42,44]. As presented in Table 1, a total of 2 studies included patients with multiple chronic diseases (including diabetes) and the other 5 studies focused solely on diabetes—4 studies focused on type 1 diabetes (T1D) [40,41,44,46], whereas 1 study included both T1D and type 2 diabetes [42]. The other studies included all adults, but some did not specify the mean age of those who participated, as shown in Table 1. With regard to the country of origin, 3 studies were conducted in the United States, with 4 out of the 7 studies originating from Israel, Macedonia, and Italy. The studies were published between 2011 and 2019.

**Table 1:** Studies on the use of social media among patients for self-care.

References and country	Target condition and age group (years) of participants	Study design and sample size	Exposure/intervention of experimental groups	Form of social media used and purpose	Outcome measures	Results
Grosberg et al [45]; Israel	DM^a^, chronic pain, hypertension, and depression (15-≥60)	Cross-sectional and prospective cohort (3 months), N=686	Active participation in a Hebrew-only website designed for chronic conditions	Discussions and blogs: *Camoni* (a Web-based social health network) for advice, consults with experts, and chats with other patients	Personal Involvement in Health Care Related to Site Use, PAM^b^	At baseline, experienced users had higher PAM scores (mean 69.3, SD 19.1; PAM level 4; *P*<.001) than new users (mean 62.8, SD 18.7; PAM level 3). At follow-up, there was a positive correlation between the frequency of visits or time spent and 3 indices of health empowerment (confidence from knowledge acquired about the disease, a sense of shared support, and personal involvement in treatment). PAM scores were higher among experienced users compared with new users (mean 62.8 vs 69.3, respectively; Z=−4.197; *P*<.001)
Iafusco et al [41]; Italy	T1D^c^ (10-18), mean age: 13.6 (SD 2.7) chat group, 14.1 (SD 2.3) control	Prospective cohort (2 years), N=396	Online group messaging once a week for 90 min	Group chatting: I Seek You program for educational purposes and social support	DQOLY^d^ and HbA_1c_^e^	The intervention group showed significant improvements in all 3 subscales of DQOLY compared with the control: impact of diabetes (mean 75, SD 7 vs mean 81, SD 14; *P*<.001), worries about diabetes (mean 27, SD 3 vs mean 49, SD 2; *P*=.001), and satisfaction with life (mean 68, SD 13 vs mean 35, SD 13; *P*<.001). No statistically significant difference (*P*=.06) was observed in HbA_1c_ values between the chat and nonchat groups
Magnezi et al [43]; Israel	DM, CVD^f^, kidney disease, spinal cord injury, depression/anxiety (20-≥65)	Cross-sectional, N=296	Active participation in a Hebrew-only website designed for chronic conditions	Discussions and blogs: *Camoni* for advice, consults with experts, and chats with other patients	Perceived Usefulness of Online Groups, PAM-13	Perceived usefulness was significantly higher in the 20-29 age group (mean 2.26, SD 1.24) than 50-64 age group (mean 1.43, SD 1.18; *P*=.04) and ≥65 age group (mean 1.38, SD 1.00; *P*<.05). PAM-13 was significantly lower in the 20-29 age group (mean 48.44, SD 21.25) compared with the 30-39 age group (mean 62.28, SD 19.78; *P*=.01) and the 50-64 age group (mean 57.50, SD 17.66; *P*<.05)
Nelakurthi et al [42]; United States	Type 1, type 2, and unspecified type DM, ≥18 (mean age 57, SD 14)	Cross-sectional, N=212	Visiting DM—specific social networking websites	DM—specific social networking websites	Following advice regarding eating habits, exercise habits, and lifestyle changes related to diabetes	Website users showed a significant correlation between offering advice and applying it to their own eating habits (*r*=0.29; *P*=.005), exercise (*r*=0.41; *P*=.001), and lifestyle modification (*r*=0.38; *P*=.001)
Newton et al [40]; United States	T1D (13-18)	RCT^g^, N=50	Standard medical care plus website participation (7 weeks)	Discussion, blogs, and group chatting: *Diabetes Teen Talk*, to discuss solutions to psychosocial problems that make compliance difficult	DQOLY, Self-Efficacy of Diabetes Self-Management, and Outcome Expectations of Diabetes Self-Management	No significant differences between the control and intervention group on Quality of Life (*P*=.63), Self-Efficacy (*P*=.53), or Negative Outcome Expectation (*P*=.31) scores. Higher positive outcome expectations on treatment conditions was in the control group compared with the intervention group (mean 44.5, SD 6.9, *P*=.03)
Petrovski et al [46]; Macedonia	T1D (11-25), mean age: noninternet group 15.2 (SD 2.9), internet group 16.4 (SD 1.9)	Cross-sectional and retrospective cohort, N=728	Participating members in a national closed Facebook group	Discussion and blogs: Facebook, better blood glucose control, and social support	HbA_1c_ (%), HbA_1c_ (mmol/mol), diabetes ketoacidosis per patient/year, severe hypoglycemia per patient/year, and total daily insulin	Significant differences in the Facebook group between HbA_1c_ (%) and HbA_1c_ (mmol/mol; mean 7.1, SD 3.2 and mean 54, SD 35, respectively) compared with the control (mean 7.6, SD 2.8 and mean 60, SD 31, respectively; *P*<.05 in both). No significant differences in other measures
Oser et al [44]; United States	T1D (≥18)	Cross-sectional, N=282	Only passive readers of T1D-related blogs with no active contribution, insulin pump use, and CGM^h^	T1D-related blogs	HbA_1c_ (%)	HbA_1c_ levels of blog users were significantly lower than nonusers (7.0% vs 7.5%; *P*=.006), blog readers on insulin pump vs blog nonusers and those not on insulin pump (7.0% vs 8.0%), and blog users using CGM vs blog nonusers not using CGM (6.9% vs 7.5%)

^a^DM: diabetes mellitus.

^b^PAM: Patient Activation Measure.

^c^T1D: type 1 diabetes.

^d^DQOLY: Diabetes Quality of Life for Youth Inventory.

^e^HbA_1c_: glycated hemoglobin.

^f^CVD:cardiovascular disease

^g^RCT: randomized controlled trial.

^h^CGM: continuous glucose monitor.

### Form of Social Media Used and Purpose

Out of the 7 studies, 4 used discussion forums or blogs, either through websites developed especially for the targeted population [40,43-45] or through commercially available platforms (eg, Facebook and I Seek You) [41,46]; 2 studies did not use a platform or website but instead evaluated respondents’ social networking site behaviors [42,44]; 2 studies used social media as a form of social support [40,46]; 3 studies assessed the usefulness of the platforms for educational purposes [41,43,45]; 2 studies used social media as a tool to improve blood sugar control through educating participants on the technicalities of blood glucose measurement and management, especially for the youth [40,46]; and 1 study assessed the accessibility and usefulness of Web-based medical information [42].

### A Description of Social Media Use and Intended Outcomes

#### Clinical Outcomes

Concerning clinical outcomes, 3 of the 7 studies reviewed reported glycated hemoglobin (HbA_1c_). Petrovski and Zivkovic [46] and Iafusco et al [41] focused on the adolescent age group, whereas Oser et al [44] targeted adults (≥18 years) with T1D. Petrovski and Zivkovic [46] sought to evaluate a Facebook group as a communication tool to interact with questions, answers, and comments to improve glucose control among adolescents and young people with T1D. Using a retrospective cohort design, Petrovski and Zivkovic [46] reported on data that were collected about Facebook users via electronic medical records and a cross-sectional analysis via social media (both Facebook and Viber). Patients from the Facebook group had a mean of 1.5 (SD 3.5) posts per day [46]. Among 728 members in their diabetes center, they found significantly lower levels of HbA_1c_ among Facebook group users compared with nonusers 1 year after joining the closed Facebook group (users mean 7.1, SD 3.2; nonusers mean 7.6, SD 2.8; *P*<.05; N=728) [46].

Iafusco et al [41] evaluated the effectiveness of a chat line for T1D education among the youth using a prospective cohort design. In contrast to the study above, Iafusco et al [41] did not find a statistically significant difference in HbA_1c_ levels between 2 groups after adjusting for therapy choice, although the differences approached significance (*P=*.05). HbA_1c_ was assessed on each participant (N=396) at baseline, year 1, and year 2 (N=193) [41]. One important consideration of this study is that children mature physically, mentally, and emotionally over the course of 2 years. It is possible that HbA_1c_ changed similarly for both groups because blood glucose control was an issue of maturity and not necessarily related to the chat line.

Oser et al [44] focused on adults with T1D to assess HbA_1c_ differences between blog readers and blog nonusers [44]. This cross-sectional study also looked at differences in technology use (insulin pump and continuous glucose monitors) in these 2 groups and self-reported HbA_1c_ differences in blog use and technology subgroups [44]. Among 214 blog readers and 68 blog nonusers who completed their survey, the authors found HbA_1c_ was lower for blog readers (7.0%) compared with blog nonusers (7.5%; *P*=.006) [44]. The difference between blog users vs blog nonusers was compared with the clinically significant difference in HbA_1c_ seen among those who used continuous glucose monitors (compared with nonusers) and insulin pump use (compared with multiple daily injections) [44]. These results show that reading and communicating through blogs with other individuals with diabetes leads to learning pertinent information and thereby is associated with lower HbA_1c_ values [44].

#### Behavioral Outcomes

Magnezi et al [43] and Grosberg et al [45], in 2 separate studies, evaluated *patient activation* (defined as a patient’s level of active participation in his or her health care) with chronic care management as a result of using social media. In particular, they examined the use of an online health-related social network called *Camoni*, a platform that was developed for individuals with a variety of chronic diseases to assist them in finding others with similar conditions [43,45]. The website provided advice about their common condition through blogs, discussion forums, online support groups, chats, and a secure channel to communicate with experts. Magnezi et al [43] included individuals with 5 chronic conditions: diabetes mellitus, CVD, renal disease, and depression/anxiety (N=296), whereas Grosberg et al [45] focused on individuals with diabetes, chronic pain, hypertension, and depression (N=696). The purpose of the studies was to evaluate the effects and benefits of participating in an online health-related social network on patient activation and to determine which variables predict the perceived usefulness of the site [43,45]. They found that the usefulness of the website was negatively correlated with age, and it was perceived as being more useful among participants who were less involved in their own care [43]. In addition, the level of activity on the website correlated with the perceived usefulness [43], and those with at least six months experience on the site had the highest patient activation scores (level 4) compared with new visitors (*P*<.001) [45]. There was a significant positive association among experienced users between both the frequency and duration of website visits and self-reported personal empowerment in health [45]. Gender differences were documented as men browsed the website for more than 30 min, whereas the average time for women was 10 to 30 min [45].

Using a cross-sectional study design, 2 separate studies conducted by Nelakurthi and colleagues [42] and Iafusco and colleagues [41] sought to evaluate the reasons behind the use of social networking sites among patients with diabetes and its impact on self-care. Nelakurthi et al [42] used surveys distributed through clinics and websites, whereas Iafusco et al [41] used a chat line moderated by a supervised physician, although it was unclear in the paper by Nelakurthi et al [42] which clinics and health websites were used and accessed by the patients. The top 2 reported reasons for the use of social networking sites were either to offer support or to share personal experiences [42]. Self-reported insulin therapy was significantly higher among users of social media (*P=*.01) [42]. Respondents were more likely to follow the advice received from the website about lifestyle changes and diabetes care compared with advice that was received from their health care provider, 69% and 65% of the time, respectively [42]. However, Iafusco et al [41] revealed that most of the patients thought that sharing HbA_1c_ readings on the group page was motivational for the other members of the group (64%) with the use of both Facebook and Viber.

#### Quality of Life and Self-Efficacy Factors

We found 2 other themes among 2 of the studies in this review: self-efficacy and quality of life. In a pilot RCT, Newton and Ashley [40] recruited adolescents (13-18 years of age) with T1D to assess the efficacy of a website, *DiabetesTeenTalk.com*, which provided blogs, chat rooms, and discussion forums to improve adherence to treatment protocols. All of the components were designed using Bandura’s self-efficacy theory [47]. Although 81 participants were recruited, 59 completed the pretests, and 50 (85%) completed the posttests at 7 weeks [40]. In addition to standard medical care, the experimental group participated in the intervention through logging into the website at least three times weekly over 7 weeks, updated their blogs, and participated in the discussion forums and chats; the control group received standard medical care only [40]. Blinding of subjects was not feasible considering the intervention. However, the assessors of outcomes were not blinded. Differences in characteristics between experimental and control patients were not compared with statistical analyses, although there appeared to be differences in age groups and gender between the intervention and control groups.

Newton and Ashley [40] assessed the effectiveness of the intervention using Diabetes Quality of Life for Youths (DQOLY), Self-Efficacy of Diabetes Self-Management, and Outcome Expectations of Diabetes Self-Management. Comparatively, Iafusco et al [41] examined DQOLY (N=396) at baseline, year 1, and year 2 (N=193). Newton and Ashley [40] found no significant differences between treatment groups on quality of life scores (*P=*.63), self-efficacy scores (*P=*.53), or negative outcome expectations (*P=*.31). However, the control group had higher positive outcome expectations (mean 48.1, SD 6.3) than those in the experimental group (mean 44.5, SD 6.9; *P=*.03) [40]. A large majority (78%) of the participants in the intervention group indicated that social support was the most helpful component of the website [40]. Iafusco et al [41] identified significant positive improvements in all subscales of DQOLY in the intervention (chat) group compared with controls who were randomly selected because they refused to participate in chat sessions [41]. At year 2, these included impact of diabetes (chat: mean 75, SD 7; nonchat: mean 81, SD 14; *P*<.001), worries about diabetes (chat: mean 27, SD 3; nonchat: mean 49, SD 2; *P=*.001), and satisfaction with life (chat: mean 68, SD 3; nonchat: mean 35, SD 13; *P*<.001) [41].

## Discussion

### Principal Findings

To our knowledge, this paper is the first to systematically review the literature for quantitative studies on the use of social media by patients with diabetes to communicate with peers for self-care. We identified 7 studies that examined the use of social media in managing various types of diabetes and reported on participants’ change in clinical outcomes, behavioral outcomes, quality of life, and self-efficacy factors as the study outcomes. The studies were diverse, utilizing various social media platforms (eg, discussion forums, blogs, and group chats), research designs and methodologies (eg, RCT, feasibility, prospective and retrospective cohort, and cross-sectional), outcomes (eg, questionnaires and clinical/laboratory measures), and patient populations (eg, adolescents, young adults). Although there is no consensus among experts on the best form of social media platform to connect patients with each other, there is a promising benefit of using Facebook groups, blogs, and mobile phone apps for connecting patients with chronic conditions to their peers.

Both commercially available and customized social media platforms were used by patients in our review. Facebook groups have been found to be a useful tool as they provide a multimodal platform to access content, deliver skills, monitor progress, and organize online and live groups [48,49]. In addition, these groups could be a useful tool for patients and their caregivers to learn about blood glucose devices and receive technological assistance. Through closed private groups, members provided assistance to the community by spreading awareness, technical assistance, and emotional support. Furthermore, members put a high level of trust in their peers and followed their advice in many health situations about lifestyle changes for their chronic conditions, although almost all patients reported no harm using Facebook [46,50]. Similarly, establishing online connections with other individuals experiencing a similar chronic condition through blogging was shown to decrease the sense of isolation and increase the sense of purpose. In addition, active engagement in blogs was shown to be associated with a higher sense of self-accountability and provided a greater opportunity for patients to gain knowledge about their conditions [51,52].

Among the studies we included in this review, users’ interactions with one another in the platforms were structured by 4 elements: (1) seeking support or encouragement from individuals with similar conditions, (2) seeking information and advice about clinical diabetes care, (3) obtaining advice about lifestyle changes, and (4) providing a sense of companionship [42,53]. Although obtaining information was the primary motive behind using these platforms rather than seeking relationships, several studies demonstrated that social support and motivation were the most helpful components of these platforms. For instance, a few studies demonstrated that most of the patients shared their last HbA_1c_ level with a social media group, which was used as a motivational and supportive tool for other patients [40,42,46]. Similarly, some were motivated to make other contributions in various forms, such as informational, technical, emotional, or financial support [51].

Our findings are consistent with a recent scoping review by Litchman et al [54] who assessed the potential or actual benefits and consequences of using a diabetes online community (DOC) by analyzing different study designs (cohort, cross-sectional, social network analysis, and text mining). They found that DOC use was highly beneficial with minimal risk or negative consequences [54]. Our review updates this earlier review, which analyzed patients’ communication with each other by focusing on quantitative studies. In addition, unlike our study that focused on peer-to-peer interactions, previous reviews have reported on studies between patients and health care providers and showed positive outcomes with using social media and improvement in patient care to provide social, emotional, or experiential support in chronic diseases [48,49].

### Potential Impact of Social Media in Diabetes

The benefit of peer-to-peer use is that social ties formed on online platforms provide support for self-care activities that can improve an individual’s perceived illness experience, a particularly difficult area to address otherwise [55-60]. Social media platforms provide social support with practical options for facilitating self-care and emotional support to those living with chronic conditions [61-63], which is preferred by patients except when information on prescription medications is needed. In addition, there is no liability to the health care provider with peer-to-peer communication. Health care providers need to assess their capacity to monitor and any potential risks before encouraging widespread use of social media as a communication tool for patients and families [46] and include the communication as a part of the patient’s health record. The American Association of Diabetes Educators emphasized in their most recent guidelines about the various benefits of online peer support, which included clinical, behavioral, psychosocial, and educational support [64]. This adds to the potential benefit of incorporating social media use for the management of chronic conditions such as diabetes mellitus.

### Consideration of Potential Risks

Accuracy and creditability of medical information obtained from social media platforms remains to be one of the primary concerns to patients. A number of studies have found that DOCs have beneficial effects with minimal risk [50,65-67]. Although there were positive results in this review with social media use overall, one should consider the risks that may emerge from using these platforms. These risks include access to misinformation, difficulty interpreting medical or scientific outcomes for the average reader, threats to individuals’ privacy, and distraction by advertisements on the blogs [57,67-69]. There are limited data on the potential negative outcomes resulting from such activities to warn against using social media with chronic conditions. In addition, there are currently no rigorous quantitative or qualitative data to support the use of social media within the domains of diagnosis or education.

### Limitations

There are limitations to be considered in our study. A systematic approach was used to select the relevant articles in the literature; however, we were unable to assess the methodological quality across studies because of the various study designs and some studies using a hybrid approach of cross-sectional surveys with cohort studies. A noted limitation is the small number of studies that fit the inclusion criteria of peer-to-peer communication for this systematic review paper. However, this strengthens the argument that many more clinical research opportunities exist in this area. In addition, because an inclusion criterion for this review paper included the specific mention of a chronic condition (ie, CVD, stroke, or diabetes), it is plausible that there may have been papers that were inadvertently excluded that did include these chronic conditions. Although some of the studies did not include a mean age, the majority of participants were adolescents or young adults, thus our conclusions cannot be generalized to older populations. Finally, this review only included studies published in the English language. Therefore, it is possible some relevant studies may have been excluded.

### Future Research

Future research opportunities and current gaps have been identified in this review. There is a clear need to conduct more rigorous RCTs on patients using social media to manage their chronic disease through peer-to-peer communication as we only identified 1 pilot study. By providing a strong evidence base for applying social media for self-care, we will be able to determine the efficacy of using these platforms. We must also improve our outreach to diverse populations (ie, age, types of chronic disease, and race/ethnicity) and geographic locations to establish generalizability. Social media interventions need to be tested with the overall goal of engaging patients, caregivers, and providers to improve health and psychosocial outcomes. Given the limited studies that were included in this systematic review paper, some questions require future research: What type of social media platforms are the most effective and feasible? Which is better in the self-care of chronic conditions: commercially available or customized social media platforms? Which populations benefit the most from the use of social media for the self-care of chronic conditions?

### Conclusions

This review contributes to our limited understanding of the impact of using contemporary social media platforms as a peer-to-peer communication tool among patients with diabetes to enhance self-care. Findings from this review may serve as a resource for researchers and clinicians to tailor their interventions in the way social media is currently used between patients and/or diversify their social media platforms according to the communities that they serve. There is a paucity of published research on social media use for peer-to-peer communication among patients with diabetes, which provides a ripe opportunity for clinicians and scientists to explore this digital means of communication among patients with chronic diseases. Social media platforms provide a cost-effective tool that may improve patient self-care and knowledge [54], thereby increasing patient activation, improving problem solving, and providing social support.

## Appendix

Multimedia Appendix 1 The search terms used to generate this review.

## Abbreviations

CVD: cardiovascular disease
CGM: continuous glucose monitor
DOC: diabetes online community
DQOLY: Diabetes Quality of Life for Youth
HbA_1c_: glycated hemoglobin
PAM: Patient Activation Measure
RCT: randomized controlled trial
T1D: type 1 diabetes
