# Supplementary material for: Patients’ Use of Social Media for Diabetes Self-Care: Systematic Review
Source: J Med Internet Res. 2020 Apr 24;22(4):e14209. doi: 10.2196/14209 (PMC7210496; doi:10.2196/14209)
Supplement: Multimedia Appendix 1 [file jmir_v22i4e14209_app1.docx]

**Embase**

("coronary artery disease" OR "myocardial ischemia" OR "acute coronary

syndrome" OR angina OR "coronary artery bypass" OR cabg OR "coronary

artery bypasses" OR "percutaneous coronary intervention" OR

"percutaneous coronary interventions" OR (coronary AND (balloon OR

angioplasty OR angioplasties OR atherectomy OR atherectomies)) OR

"diabetes mellitus" OR diabetes OR hyperglycaemia OR hyperglycemia OR hyperinsulinism OR "intracranial haemorrhage" OR

"intracranial haemorrhages" OR "intracranial hemorrhage" OR

"intracranial hemorrhages" OR "stroke" OR stroke OR "brain hemorrhage"

OR "brain haemorrhage" OR "brain hemorrhages" OR "brain haemorrhages")

AND

(facebook OR twitter OR tweet OR youtube OR blog OR blogs OR

blogger OR bloggers OR blogging OR instagram OR pinterest OR "google

plus" OR "google+" OR whatsapp OR "whats app" OR "flickr" OR "myspace"

OR "livejournal" OR "linkedin" OR "orkut" OR "wordpress" OR "tumblr" OR "skype" OR upstream OR illuminate OR WeChat OR QQ OR QZone OR Weibo OR Line OR Douban OR Meipai OR KakoTalk OR KakoStory OR Naver OR NaverBlog OR VKontakte OR Odnoklassniki OR Reddit OR Askfm OR Meetup)

AND

("social media" OR "social network" OR

"interpersonal communication" OR internet OR "mass communication"/exp

OR "patient to patient" OR "patient-patient")

**PubMed:**

("coronary artery disease" OR "myocardial ischemia" OR "acute coronary syndrome" OR angina OR "coronary artery bypass" OR cabg OR "coronary artery bypasses" OR "percutaneous coronary intervention" OR "percutaneous coronary interventions" OR (coronary AND (balloon OR angioplasty OR angioplasties OR atherectomy OR atherectomies)) OR "diabetes mellitus" OR diabetes OR hyperglycaemia OR hyperglycemia OR hyperinsulinism OR "intracranial haemorrhage" OR "intracranial haemorrhages" OR "intracranial hemorrhage" OR "intracranial hemorrhages" OR "stroke" OR stroke OR "brain hemorrhage" OR "brain haemorrhage" OR "brain hemorrhages" OR "brain haemorrhages")

AND

(facebook OR twitter OR tweet OR youtube OR blog OR blogs OR blogger OR bloggers OR blogging OR instagram OR pinterest OR "google plus" OR "google+" OR whatsapp OR "whats app" OR "flickr" OR "myspace" OR "livejournal" OR "linkedin" OR "orkut" OR "wordpress" OR "tumblr" OR "skype" OR upstream OR WeChat OR QQ OR QZone OR Weibo OR Line OR Douban OR Meipai OR KakoTalk OR KakoStory OR Naver OR NaverBlog OR VKontakte OR Odnoklassniki OR (Classmates AND Russia) OR Reddit OR Askfm OR Meetup)

AND

("social media" OR "social network" OR "interpersonal communication" OR internet OR "mass communication" OR "patient to patient" OR "patient-patient")

Use the PubMed search in both Web of Science and CINAHL.
